# New Insights on Phytoplankton Morpho-Functional Traits

**DOI:** 10.3390/microorganisms11061545

**Published:** 2023-06-10

**Authors:** Silvia Pulina, Cecilia Teodora Satta

**Affiliations:** Aquatic Ecology Group, Department of Architecture, Design and Urban Planning, University of Sassari, Via Piandanna 4, 07100 Sassari, Italy; ctsatta@uniss.it

The pelagic environment is characterized by a great spatial and temporal heterogeneity. For phytoplankton, this heterogeneity encompasses the availability and distribution of resources, the direction and intensity of water movements, and grazer composition and activity [1]. The considerable diversity in phytoplankton morphology represents a successful adaptive strategy designed to cope with this pelagic environmental and biological variability [2]. There is a robust connection between phytoplankton cell morphology and physiological and metabolic processes [3,4,5,6,7]. In this regard, the morpho-functional traits of phytoplankton have been recognized as powerful tools to explain the development of specific phytoplankton groups in defined environmental conditions [8,9]. Despite this, improving our knowledge of phytoplankton morpho-functional trait distribution along natural environmental gradients is still challenging due to the overlap and interaction of environmental and biological forces in aquatic environments. In addition, the extent of phytoplankton intraspecific morphological variability and its role in community responses to environmental changes remain almost unknown.

For this Special Issue titled “New Insights on Phytoplankton Morpho-Functional Traits”, we collected both experimental laboratory and field studies from marine and freshwater ecosystems. Our aim is to provide novel information on phytoplankton responses to interactions between multiple stressors, focusing on phytoplankton morpho-functional trait diversity at both inter- and intraspecific levels.

Paul et al. [10] explored the combined effects of elevated temperatures and CO_2_ on a natural phytoplankton community from the Baltic Sea, dividing phytoplankton into edible and inedible cell size classes for mesozooplankton grazers. They showed how the composition and dominance of specific phytoplankton size classes and groups can help forecast how temperate summer plankton communities will respond to complex climate changes. They also underlined the importance of size-trait-based analyses to distinguish between indirect responses from the edible group via zooplankton grazing and direct responses from the inedible group.

Charalampous et al. [11] designed a mesocosm experiment to study a natural phytoplankton community from the Baltic Sea. The plankton were first subjected to mesozooplankton grazing to manipulate size structure, followed by nutrient addition and depletion. Their findings highlighted that the mean cell size of a taxonomically complex phytoplankton community can be used as an indicator trait to predict phytoplankton responses to sequential environmental changes.

By examining long-term monitoring data collected by 80 stations located in the coastal northern Baltic Sea, Lethinen et al. [12] explored potential connections between the morpho-functional composition of phytoplankton and global climate change by analyzing physical features of the environment, water quality features by analyzing catchment change, and nutrient availability using nutrient loading. Their regionality analysis demonstrated that traits should be calculated in both absolute terms (biomass) and proportions (share of total biomass) to better understand phytoplankton community changes and to potentially supplement environmental status assessments.

In an in situ experiment, Le Noac’h et al. [13] manipulated water column stratification in Croche Lake, Quebec, a temperate lake with multiple basins. The group investigated how spatial overlap among major phytoplankton groups relates to overall taxonomic and morpho-functional community diversity, accounting for time-varying changes in environmental (thermal stratification) and biotic (zooplankton grazing) variables. They demonstrated that spatial overlap among species was related to greater functional diversity in resource acquisition and morphological traits, indicating that forced coexistence enabled niche differentiation along trait axes to alleviate interspecific competition.

Titocci et al. [14] performed feeding trials in laboratory microcosms with size-fractionated freshwater phytoplankton and two different consumer types: the cladoceran *Daphnia longispina*, as a generalist unselective filter feeder, and the calanoid copepod *Eudiaptomus* sp., as a selective feeder. The authors investigated alterations in phytoplankton morpho-functional trait distribution caused by zooplankton grazing with contrasting food size preferences and feeding behaviors. In doing so, they elucidated phytoplankton community responses to herbivore grazing with respect to composition, size, and shape distribution.

Hamer et al. [15] experimentally modeled a community consisting of two morpho-functionally different marine phytoplankton species, considering nine genotypes each of *Emiliania huxleyi* and *Chaetoceros affinis*, cultivated separately and together under different fluctuation and nutrient regimes. They detected significant intraspecific differences in *C. affinis* cell size and in the *E. huxleyi* maximum nutrient uptake rate (V_max_) and demonstrated that the intraspecific diversity of one species can be affected by the presence of another. The authors concluded that the coexistence of species might play an important role in the maintenance of intraspecific morpho-functional diversity.

The results derived from this Special Issue confirm the value of morpho-functional trait-based approaches for evaluating phytoplankton responses to environmental change, with a particular focus on grazing. Further studies are certainly needed, but this Special Issue provides the knowledge needed to predict the effects of ongoing climatic change on phytoplankton dynamics and its relative consequences for aquatic ecosystems.
